# A High-Sodium Diet Modulates the Immune Response of Food Allergy in a Murine Model

**DOI:** 10.3390/nu13113684

**Published:** 2021-10-20

**Authors:** Zheying Liu, Shih-Kuan Li, Chih-Kang Huang, Ching-Feng Huang

**Affiliations:** 1 Emergency Department, Department of Emergency and Critical Medicine, Wan Fang Hospital, Taipei Medical University, Taipei City 11696, Taiwan; 102230@w.tmu.edu.tw; 2 Department of Emergency, School of Medicine, College of Medicine, Taipei Medical University, Taipei City 11031, Taiwan; 3 Department of Pediatrics, Wan Fang Hospital, Taipei Medical University, Taipei City 11696, Taiwan; 4 Division of Pediatric Gastroenterology, Hepatology and Nutrition, Department of Pediatrics, Taipei Veterans General Hospital, Taipei City 11217, Taiwan; calm2577@gmail.com (S.-K.L.); follyfolly25@yahoo.com.tw (C.-K.H.); 5 Department of Pediatrics, Yonghe Cardinal Tien Hospital, New Taipei City 23445, Taiwan; 6 Department of Pediatrics, Taipei Veterans General Hospital, Taoyuan Branch, Taoyuan City 33052, Taiwan; 7 National Defense Medical Center, School of Medicine, Taipei City 11490, Taiwan

**Keywords:** food allergy, ovalbumin, IgE, high salt diet, Th2 response

## Abstract

Mounting evidence demonstrates that a high-salt diet (HSD) not only affects hemodynamic changes but also disrupts immune homeostasis. The T helper 17 (Th17) and regulatory T cells (Tregs) are susceptible to hypersalinity. However, research on the influence of sodium on Th2-mediated food allergies remains scarce. We aimed to investigate the effect of dietary sodium on the immune response to food allergies. Mice maintained on an HSD (4% NaCl), low-salt diet (LSD; 0.4% NaCl), or control diet (CTRL; 1.0% NaCl) were orally sensitized with ovalbumin (OVA) and a cholera toxin (CT) adjuvant, and then subjected to an intragastric OVA challenge. OVA-specific immunoglobulin G (IgG), IgG1, IgG2a, and IgE antibodies were significantly higher in the HSD group than in the CTRL group (*p <* 0.001, *p* < 0.05, *p* < 0.01, and *p* < 0.05, respectively). Mice on HSD had significantly higher interleukin (IL)-4 levels than the CTRL group (*p* < 0.01). The IL-10 levels were significantly lower in the HSD group than in the CTRL group (*p* < 0.05). The serum levels of interferon-γ (IFN-γ), sodium, and chloride did not differ among the three groups. This study indicates that excessive salt intake promotes Th2 responses in a mouse model of food allergy.

## 1. Introduction

Globalization has caused rapid changes in people’s eating habits, leading to an increased consumption of processed and packaged foods, and a lifestyle that is based on a high-salt diet (HSD). Excessive salt consumption can pose a threat to human health. High dietary salt intake has been linked to many well-recognized diseases, such as cardiovascular complications, hypertension, and metabolic syndromes [1]. Mounting evidence on the effects of HSD has demonstrated that it not only mediates hemodynamic changes but also disrupts immune homeostasis. It is well established that excessive salt augments the differentiation of naïve T cells into T helper 17 cells (Th17), resulting in the onset and exacerbation of autoimmune conditions in animal models of multiple sclerosis, lupus nephritis, rheumatoid arthritis, and Crohn’s disease [2,3,4]. There is a fine balance between Th17 and regulatory T cells (Tregs). Furthermore these T-helper subsets are reciprocally regulated, which enables the transition between pro- and anti-inflammatory states [5]. Therefore, the enhanced differentiation of Th17 cells after exposure to high concentrations of salt may further dampen Treg phenotypes. Moreover, excessive salt was shown to exert a direct effect on the suppressive functions of Tregs and exacerbate experimental graft-versus-host diseases [6]; Th2 and Tregs also share such a relationship. A change in the equilibrium between allergen-specific Th2 and Treg cells can either result in the development of allergic diseases or in the recovery from allergy [7,8]. A previous study demonstrated that the failure to induce oral tolerance, or the breakdown of oral tolerance as a result of the impaired generation or functioning of the suppressive Tregs, could contribute to food allergy [9]. Newly discovered evidence revealed that the epigenetic modifications, caused by decreased or increased levels of histone acetylation at key Th cell loci, contributed to the allergy to cow’s milk protein or the allergy-protective effect of raw milk [10,11]. While it is well known that sodium is an immunomodulator of Th17 cells and Tregs [12], our understanding of the direct effect of sodium on Th2-dependent allergic diseases, such as food allergies, remains scarce.

The expression of food allergies is multifactorial and is affected by the genetic background of an individual, environmental factors, and interactions between the genome and environment, including the epigenetic effects. The prevalence of food allergies has been constantly increasing over the last three decades [13]. As evidenced by the epidemiologic studies, up to 10% of the population is affected by food allergies [14]. The present standards for treating food allergy include allergen avoidance and immediate access to medication in the event of anaphylaxis [13]. These are relatively safe and effective measures for controlling symptoms but not for curing the disease. Food allergies are characterized by an overriding Th2 response. The increasing prevalence of food allergies, together with a rise in human urbanization may indicate a correlation between the two. Moreover, urbanization leads to changes in lifestyle and diet is one of the most rapid of these changes. The limited evidence on the impact of dietary components, such as food additives and vitamin D n-3/n-6 polyunsaturated fatty acids, on the homeostasis of the immune system suggests that these components may hinder or facilitate the development of food allergies [15,16,17]. However, the impact of HSDs or low-salt diets (LSDs) on food allergy has not yet been ascertained. Since sodium chloride (NaCl) has been shown to affect immune homeostasis, we hypothesized that a high salt intake might have an effect on food allergies. Here, we aimed to investigate the effect of dietary salt intake on the immune response in a mouse model of food allergy.

## 2. Materials and Methods

### 2.1. Animals and Ethics Statement

Eight-week-old female BALB/c mice were purchased from the National Animal Center (Taipei, Taiwan). All mice were housed in cages under conventional conditions of controlled temperature and relative humidity with a regular 12 h light/dark cycle in the Animal House of the National Defense Medical Center (Taipei, Taiwan). All animal experiments were approved by the Institutional Animal Care and Use Committee of the National Defense Medical Center (Ethical approval number: IACUC-13-121).

### 2.2. Antigen Preparation

Ovalbumin (OVA) grade V was acquired from Sigma-Aldrich (St. Louis, MO, USA). Cholera toxin (CT; Calbiochem, San Diego, CA, USA) was used as an adjuvant. Briefly, 360 mL of 1 mg/mL OVA and 90 μL of 2 mg/mL CT were dissolved in 9 mL phosphate-buffered saline (PBS).

### 2.3. Experimental Design

After an acclimatization period of one week, the mice were randomly divided into the following three groups: HSD, LSD, and control (CTRL) (*n* = 6 mice/group). In the HSD group, naïve mice were exposed to HSD (TestDiet^®^, St. Louis, MO, USA) that was supplemented with 4% NaCl. In the LSD group, naïve mice were administered chow with 0.4% NaCl (TestDiet^®^), whereas mice from the control group were fed a normal salt diet (TestDiet^®^) containing 1.0% NaCl. One-percent, NaCl-containing water was provided to mice from the HSD group, and distilled water was provided to the mice in the LSD and control groups. All mice were maintained on a specialized rodent diet and water ad libitum for 10 weeks (weeks 0 to 10).

**Figure 1:**
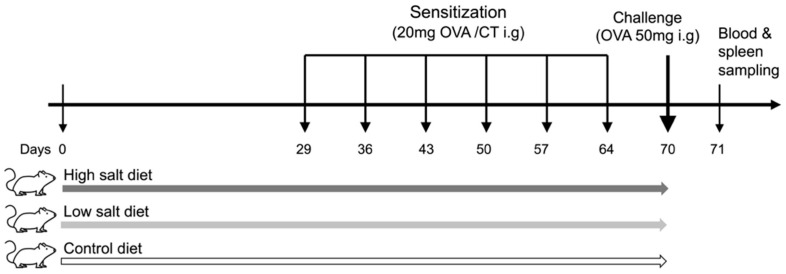
Experimental protocol. Three different experimental protocols were used for priming. Mice were fed a high- or low-salt diet or control diet ad libitum for 10 weeks. After 4 weeks of exposure to different sodium concentrations, all mice were intragastrically sensitized with 20 mg of ovalbumin (OVA) and 10 μg of cholera toxin (CT) once every week, for six weeks. After sensitization, mice were challenged with 50 mg of OVA via intragastric gavage. All mice were euthanized for blood and spleen sampling 1 day after the OVA challenges. Ovalbumin: OVA, cholera toxin: CT, intragastrically: i.g.

All mice were first sensitized and thereafter challenged with OVA intragastrically. Briefly, the mice were intragastrically administered 20 mg of OVA in the presence of 10 μg of CT adjuvant, which was suspended in 500 μL of PBS, once a week for six weeks. In the week after the last sensitization, mice were challenged with 50 mg OVA suspended in 200 μL of PBS via intragastric gavage after overnight fasting. All mice were euthanized one day after the OVA challenge, and blood and spleen samples were harvested for further analyses. The experiments were performed in duplicates to obtain representative data. The experimental scheme is illustrated in Figure 1.

### 2.4. Measurement of OVA-Specific Immunoglobulin G (IgG), IgG1, and IgG2a Antibodies

Blood samples were collected after challenge. The levels of OVA-specific IgG, IgG1, and IgG2a were measured using enzyme-linked immunosorbent assays (ELISA) (R&D Systems, Minneapolis, MN, USA), as described previously [18]. Briefly, microtiter plates (96 wells; Nunc, Kamstrup, Roskilde, Denmark) were coated overnight at 4 °C with 100 μL of OVA (100 μg/mL) in 0.05 M sodium carbonate (pH 9.6). On the next day, the plates were blocked with 3% skimmed milk in PBS-Tween 20 by incubation for 1 h. Serum samples (1/30–1/1000) and standards (pooled hyperimmune sera after monthly treatment with OVA emulsified in complete Freund’s adjuvant) were added to the plates in duplicates. The plates were then incubated for 5 h at room temperature. An amount of 100 mL horseradish peroxidase conjugated with goat anti-mouse IgG (1/4000; Jackson, West Grove, PA, USA), IgG1 or IgG2a (1/4000 for both; SBA, Birmingham, AL, USA) were added to each well and incubated overnight at 4 °C. Between each incubation, the plates were washed with PBS containing 0.05% Tween 20. Color was developed by adding orthophenyleldiamine (0.5 mg/mL; Sigma) in citrate-carbonate buffer containing 0.015% hydrogen peroxide and incubated in the dark at room temperature. Finally, the reaction was stopped with 4 N sulfuric acid. A SPECTRAmax 250 reader (Molecular Devices, Sunnyvale, CA, USA) was used to measure the absorbance at 492 nm, and unknowns were interpolated.

### 2.5. Measurement of OVA-Specific IgE Antibody

OVA-specific IgE antibodies in mouse serum were detected by in vivo passive cutaneous anaphylaxis (PCA) assay, as described previously [18]. Briefly, Sprague Dawley rats were purchased from the Animal Center, National Yang-Ming University, Taipei, Taiwan. Aliquots (100 μL) of 2-fold dilutions of mouse serum samples (1/50–1/800) were intradermally injected into the rats. They were then challenged after 48 h via an intravenous injection of 2 mg of OVA and 5 mg of Evans Blue in 1 mL PBS. Thirty minutes after the challenge, the rats were sacrificed and the diameter of the cutaneous reaction was measured. A positive IgE response was recorded if the challenge resulted in a blue lesion ≥5 mm on the skin of 50% or more recipient animals. The antibody titer was expressed as the highest dilution of the serum sample to give a positive PCA reaction.

### 2.6. Analysis of Cytokine Production in OVA-Stimulated Spleen Cells

Cytokine production in spleen cells was analyzed as described previously [18]. A day after the oral OVA challenge, spleen cells from the six BALB/c mice of each group were gently crushed and cultured (2 × 10^6^ cells per well) in 24-well flat-bottomed microtiter plates (1 mL per well; Costar, Cambridge, MA, USA) with OVA, in duplicates, and in complete Roswell Park Memorial Institute 1640 (RPMI) medium (1 mg/mL) supplemented with 10% fetal calf serum and antibiotics. Culture supernatants were harvested after 1–3 days of incubation. The levels of interleukin (IL)-4, IL-10, and interferon-γ (IFN-γ) from the harvested supernatants were measured using sandwich, enzyme-linked immunosorbent assay (ELISA) kits (e-Bioscience, San Diego, CA, USA) according to the manufacturer’s instructions.

### 2.7. Statistical Analysis

All the experiments were performed in duplicates. Experimental data were expressed as box-and-whisker plots with individual data points. Statistical comparisons between the two groups were made by the non-parametric Mann–Whitney U-test. Differences were considered significant at *p* < 0.05. Analysis was performed using GraphPad Prism version 9.1.1 (223) for Mac (GraphPad Software, San Diego, CA, USA).

## 3. Results

### 3.1. HSD Induces High Levels of OVA-Specific Serum IgG, IgG1, IgG2a, and IgE in Mice

**Figure 2:**
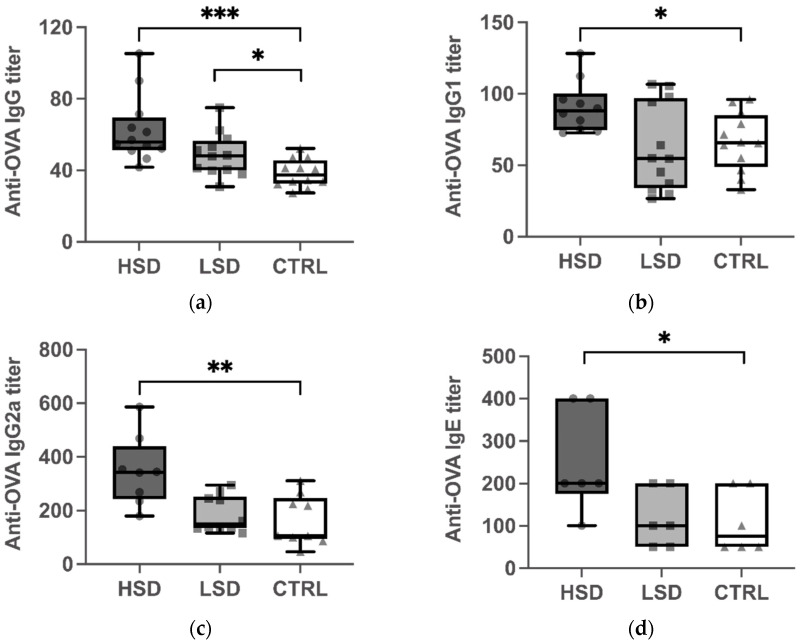
Effect of different concentrations of dietary sodium on the production of OVA-specific immunoglobulin G (IgG) (**a**), IgG1 (**b**), IgG2a (**c**), and IgE (**d**) antibodies in mice with OVA-induced food allergy. Serum was collected after challenge with the OVA antigen. IgG (**a**), IgG1 (**b**), and IgG2a (**c**) levels were examined by the enzyme-linked, immunosorbent assay (ELISA) and IgE (**d**) levels by the in vivo passive cutaneous anaphylaxis (PCA) test. Data are expressed as box-and-whisker plots with individual data points. The boxes represent the inner quartiles value range with the median indicated as black line. The whiskers represent minimum to maximum interval. * *p* < 0.05, ** *p* < 0.01, and *** *p* < 0.001. High-salt diet: HSD, Low-salt diet: LSD, Control diet: CTRL.

To investigate the impact of salt intake on the humoral response in sensitized mice, we measured plasma levels of OVA-specific IgG, IgG1, IgG2a, and IgE antibodies after an oral challenge with the OVA-antigen (Figure 2). OVA-specific IgG, IgG1, IgG2a, and IgE levels were significantly higher in the HSD group than in the CTRL group (*p* < 0.001 for Figure 2a; *p* < 0.05 for Figure 2b; *p* < 0.01 for Figure 2c; *p* < 0.05 for Figure 2d). Conversely, there were no statistical differences between the levels of OVA-specific IgG1, IgG2a, and IgE serum antibodies between the LSD and CTRL groups.

### 3.2. High IL-4 and Low IL-10 Production in Splenocytes of Mice Maintained on HSD

**Figure 3:**
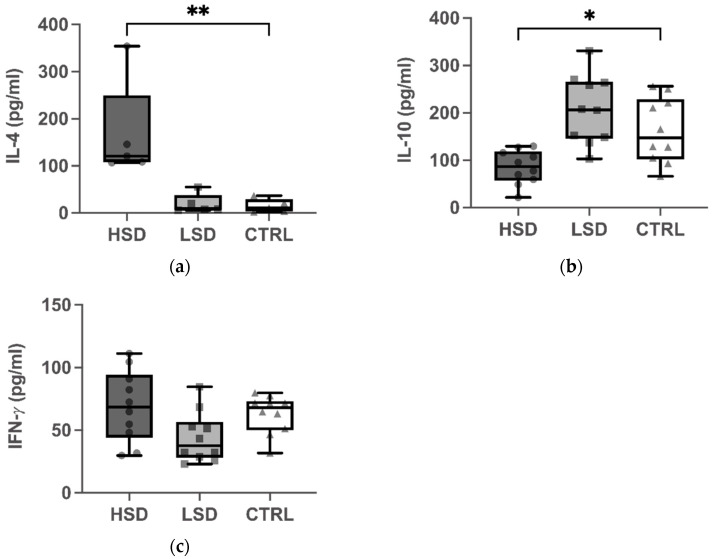
Effect of different concentrations of dietary sodium on the production of cytokines by splenocytes. Splenocytes were isolated from the spleens after OVA challenge and incubated in culture medium containing fetal bovine serum (FBS) and OVA for 1 to 3 days. Interleukin (IL)-4 (**a**), IL-10 (**b**), and interferon- *γ* (IFN-*γ*) (**c**) were measured by sandwich ELISA. Data are expressed as box-and-whisker plots with individual data points. The boxes represent the inner quartiles value range with the median indicated as black line. The whiskers represent minimum to maximum interval. * *p* < 0.05 and ** *p* < 0.01. High-salt diet: HSD, Low-salt diet: LSD, Control diet: CTRL.

Next, we evaluated the effect of sodium intake on the cytokine production in the spleens of mice with food allergies. The concentration of IL-4 was significantly higher in the HSD group than in the CTRL group, after the stimulation with OVA (*p* < 0.01 for Figure 3a). In contrast, the IL-10 levels were markedly lower in the HSD group than in the CTRL group (*p* < 0.05 for Figure 3b). No significant difference were observed in the IFN-*γ* levels between the HSD and CTRL groups (Figure 3c). On the contrary, LSD did not significantly change IL-4, IL-10, and IFN-*γ* production in the spleen cells of OVA-sensitized mice.

### 3.3. HSD Causes No Change in the Serum Levels of Sodium and Chloride

**Figure 4:**
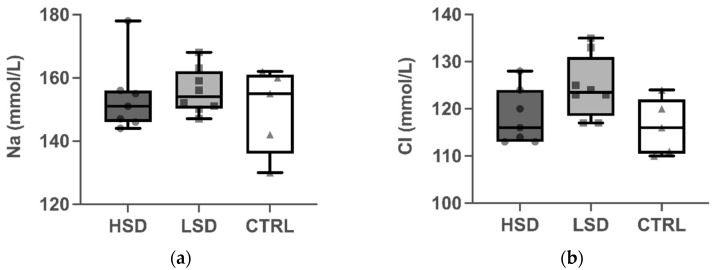
Effect of different concentrations of dietary sodium on the levels of serum sodium (Na; (**a**)) and chloride (Cl; (**b**)). Mice were fed a high- or low-salt diet, or a control diet ad libitum for 10 weeks. Blood samples were collected 1 day after administering the specialized diet. Data are expressed as box-and-whisker plots with individual data points. The boxes represent the inner quartiles value range with the median indicated as black line. The whiskers represent minimum to maximum interval. High-salt diet: HSD, Low salt diet: LSD, Control diet: CTRL.

To determine the effect of the different salt concentrations on electrolyte homeostasis, plasma concentrations of sodium (Na) and chloride (Cl) were evaluated after the administration of a special salt diet for 10 weeks (Figure 4). Dietary salt had no effect on plasma levels of Na and Cl for all mice from the three groups.

## 4. Discussion

HSD is suggested to be an environmental factor that modulates T cell differentiation, and which may promote the differentiation of naïve T cells into effector cells that are associated with autoimmune disease, such as Th17 cells. Our present knowledge about the effect of sodium on Th2-mediated allergic diseases, such as food allergies, is largely limited. In this study, mice from the HSD and LSD groups were administered a diet supplemented with 4% or 0.4% NaCl, and compared to mice from the control group, which received chow containing 1% NaCl. All mice were sensitized with OVA and the CT adjuvant, and thereafter subjected to an intragastric challenge. We observed a significant increase in the levels of serum OVA-specific IgG1, IgE, and splenic IL-4, and a significant decrease in the splenic IL-10 levels of mice from the HSD group (Figure 2 and Figure 3). This indicated that the intake of a diet supplemented with excessive dietary sodium altered immune homeostasis and promoted Th2 immune responses in a mouse model for OVA-induced food allergy. This is the first report to provide experimental evidence for the effect of sodium exposure on food allergy. Our results provide evidence that, in addition to its well-described effect on the induction of proinflammatory Th17 cells and the abrogation of the suppressive capacity of Tregs, excessive salt intake skews T cell differentiation towards Th2 responses in a mouse model of food allergy.

Food allergy is an immunologically aberrant reaction to food allergens, mainly proteins. The immunoglobulins, IgG1 and IgE, are both associated with Th2-type immune responses. Numerous animal studies have already demonstrated that OVA, as a common allergen, can increase the production of OVA-specific IgG1 and IgE serum antibodies after sensitization, thereby suggesting the induction of a Th2 response [19,20,21]. The reports on the role of NaCl in food allergies are sparse and controversial. One study reported that cultivating murine CD4^+^ T cells, in the presence of hypertonic NaCl (40 mM), showed impaired Th2 cell differentiation [22]. In contrast, another recently published pilot study demonstrated that hypersalinity enhanced the production of signature Th2 cytokines, namely IL-4 and IL-13, in memory T cells from healthy human donors [23]. Furthermore, NaCl could facilitate the differentiation of human and mouse-derived naïve T cells into Th2 cells, independent of Th2-polarizing cytokines, via the osmosensitive transcription nuclear factor of activated T-cells 5 (NFAT5) and the enzyme serum/glucocorticoid regulated kinase 1 (SGK-1) [23]. In line with this previously published data, our study revealed that the levels of serum OVA-specific IgG1, IgE, and splenic IL-4 were significantly elevated in mice from the HSD group, suggesting that a high-sodium intake potentiated dysregulated immune responses and directly enhanced the Th2 differentiation in mice. Anti-OVA IgG levels are relatively non-specific parameters and represent indicators for frequent OVA exposure [24]. Previous studies showed that some strains of mice failed to mount a significant IgE antibody response to ovalbumin despite the presence of an adjuvant [25]. In this study, we measured both IgE and IgG to confirm the successful elicitation of an allergen’s immune response to the ovalbumin. Our findings showed that anti-OVA IgG levels were significantly higher in the HSD and LSD group than in the CTRL groups. The markedly elevated levels of IgG and IgE indicated a potentiated allergic response in the HSD group. In the contrary, OVA-specific IgG was significantly reduced in the CTRL group compared to the LSD group. However, OVA-specific IgE, IgG1 and IgG2a did not significantly differ. It is possible that there may be other IgG subclasses that may have contributed to the decrease in OVA-specific IgG to induce the potential tolerance in the CTRL group. However, due to no difference in the OVA-specific IgE, IgG1, and IgG2a between LSD and the CTRL group, further studies will be needed to investigate the effect of low salt diet on the food allergy. Based on these results, we suggest that the intake of chow supplemented with high sodium upregulates antigen-specific, Th2-related antibody responses in mice.

Functional Tregs are important for maintaining tolerance to innocuous exogenous antigens and self-antigens. IL-10 is a key cytokine secreted by Tregs that can limit T cell responses. In addition to the existing evidence that Tregs limits the pathogenesis of autoimmune diseases and prevents allograft rejection, accumulating evidence suggests that Tregs might play a critical role in controlling the expression of allergic diseases. In a mouse model of peanut allergy, CD4^+^ CD25^+^ T cell-depleted mice showed impaired oral tolerance upon the exposure to peanuts and induced an IgE-mediated food hypersensitivity response after an oral challenge [26]. In the case of rare diseases, such as X-linked autoimmunity–allergic dysregulation syndrome (XLAAD)/immunodysregulation polyendocrinopathy enteropathy X-linked (IPEX), the patients lack CD25^+^ Tregs and could develop severe eczema, eosinophilia, elevated IgE, and food allergies, which indicated that Tregs were crucial for the development of allergic diseases [27]. Our results showed a significant decrease in the IL-10 levels of mice from the HSD in comparison to the control, suggesting that the function of Tregs was impaired. Moreover, this functional impairment of Tregs could further promote a type 2 immune response due to the loss of suppression, which was also observed in the present study with the markedly elevated IgG1, IgE, and IL-4 levels in the HSD group. This was consistent with previous studies, which showed that excessive dietary salt had a negative effect on the suppressive function of Tregs via inducing SGK1-mediated FOXO1 phosphorylation, which further led to the destabilization of FOXP3 [6,28]. The low IL-10 levels in our study implied that excessive sodium may indirectly skew Th2 polarization by attenuating the Treg function in the mouse model of food allergy. Existing studies demonstrated how epigenetic mechanisms affected Tregs by decreased levels of histone acetylation in the allergy to cow’s milk [11]. Whether high-sodium condition also plays a role in epigenetic modifications by regulating the severity of food allergy should be investigated in follow-up studies.

The results regarding the effect of salt on the differentiation of Th1 cells are barely comparable with previous studies. A study in mice showed that HSD had no effect on Th1 cell differentiation [22]. The levels of IgGa2 in OVA-allergy mouse models varied greatly between studies. Previous studies reported that CT could induce a weak Th1 response characterized by elevated IgGa2 levels [29,30,31]. In line with previous studies, we observed a significant increase in IgG2a levels in the HSD group, which suggested that HSD affected the frequency of Th1 cells. However, there was no significant change in IFN-γ levels between mice from the HSD and control groups. The inability of high salt to induce IFN-γ production is in accordance with the previous findings and could be explained by the low levels of SGK1 expression in the Th1 cells [3].

Concerning the effect of LSD on the adaptive immune response, we did not observe any significant influence on Th1- or Th2-related antibodies and cytokines in our study. This suggested that HSD could have a detrimental effect on human health in many aspects, while the extreme restriction of salt intake may not have a definite benefit against food allergy. With regard to the effect of the sodium on electrolyte homeostasis, we found no differences in plasma levels of sodium and chloride from the three groups. The accumulating evidence has shown that sodium could distribute at a different concentration throughout the human body and may reach hypersalinity in the interstitium regardless of the circulating levels [28,32,33]. A previous study demonstrated that sodium was concentrated in the colons of mice on an HSD, indicating the direct impact of salt within the colon [34]. In the tissue microenvironment, sodium could regulate the differentiation and function of immune cells via modulating signaling pathways and contributing to protective or proinflammatory immunity.

There are several limitations in the present study. First, it was performed in an animal model and the numbers of animals were limited. We selected a small sample size because of the effect of a high-salt diet on food allergy was evaluated in vivo for the first time in the present study, and thus the initial intention was to gather basic evidence regarding the use of this experimental protocol in more complex experimental designs. Further research was necessary to investigate involving larger groups of animals to validate reproducibility. Second, whether HSD exacerbated the severity of the clinical manifestations of food allergy was not investigated in the current study and should be investigated in the follow-up study. Moreover, we measured Th cell-dependent immune responses but did not analyze the types of differentiated T cells which may provide further information on the effect of sodium on T cell polarization.

## 5. Conclusions

In conclusion, our findings suggest that HSD may play an essential role in type 2 immune responses in a given microenvironment and extend the pre-existing evidence on the ability of HSD to affect type 2 driven diseases, such as food allergies. Our findings provide putative evidence that, although it warrants further research, controlling the intake of dietary salt by targeting NaCl-induced signaling may be a promising therapeutic strategy for improving adjuvant therapy in patients with food allergy.

## Data Availability

The data that support the findings of this study are available from the corresponding author upon reasonable request.
